# Early innate cell interactions with *Mycobacterium tuberculosis* in protection and pathology of tuberculosis

**DOI:** 10.3389/fimmu.2023.1260859

**Published:** 2023-10-27

**Authors:** Poornima Sankar, Bibhuti Bhusan Mishra

**Affiliations:** Department of Immunology and Microbial Disease, Albany Medical College, Albany, NY, United States

**Keywords:** *Mycobacterium tuberculosis*, host-pathogen interactions, alveolar epithelial cells, granulocytes, inflammation, innate immunity, macrophages, pattern recognition receptors

## Abstract

Tuberculosis (TB) remains a significant global health challenge, claiming the lives of up to 1.5 million individuals annually. TB is caused by the human pathogen *Mycobacterium tuberculosis* (Mtb), which primarily infects innate immune cells in the lungs. These immune cells play a critical role in the host defense against Mtb infection, influencing the inflammatory environment in the lungs, and facilitating the development of adaptive immunity. However, Mtb exploits and manipulates innate immune cells, using them as favorable niche for replication. Unfortunately, our understanding of the early interactions between Mtb and innate effector cells remains limited. This review underscores the interactions between Mtb and various innate immune cells, such as macrophages, dendritic cells, granulocytes, NK cells, innate lymphocytes-iNKT and ILCs. In addition, the contribution of alveolar epithelial cell and endothelial cells that constitutes the mucosal barrier in TB immunity will be discussed. Gaining insights into the early cellular basis of immune reactions to Mtb infection is crucial for our understanding of Mtb resistance and disease tolerance mechanisms. We argue that a better understanding of the early host-pathogen interactions could inform on future vaccination approaches and devise intervention strategies.

## 1 Introduction

*Mycobacterium tuberculosis* (Mtb) remains the leading cause of death by a microbial pathogen in the world today even after being around for centuries. Mtb has the ability to remain dormant in the lungs of infected individuals by establishing latent TB infection (LTBI). This can later become active, disseminate to other parts of the body and progress to active TB disease (ATB) in some infected individuals. *Mtb* encounters a myriad of host cells after entering the lung airways. Early encounters of host cells with the bacteria decide whether the bacterium can either be eliminated by the immune system, contained, or replicate uncontrollably. The early bacterial interaction with the type of host cell determines the fate of an infection, though other factors such as genetical background of both the host and bacterial strain, exposure to environmental mycobacteria, parasites and infection does play a critical role in shaping the eventual outcome of infection.

**Figure 1:**
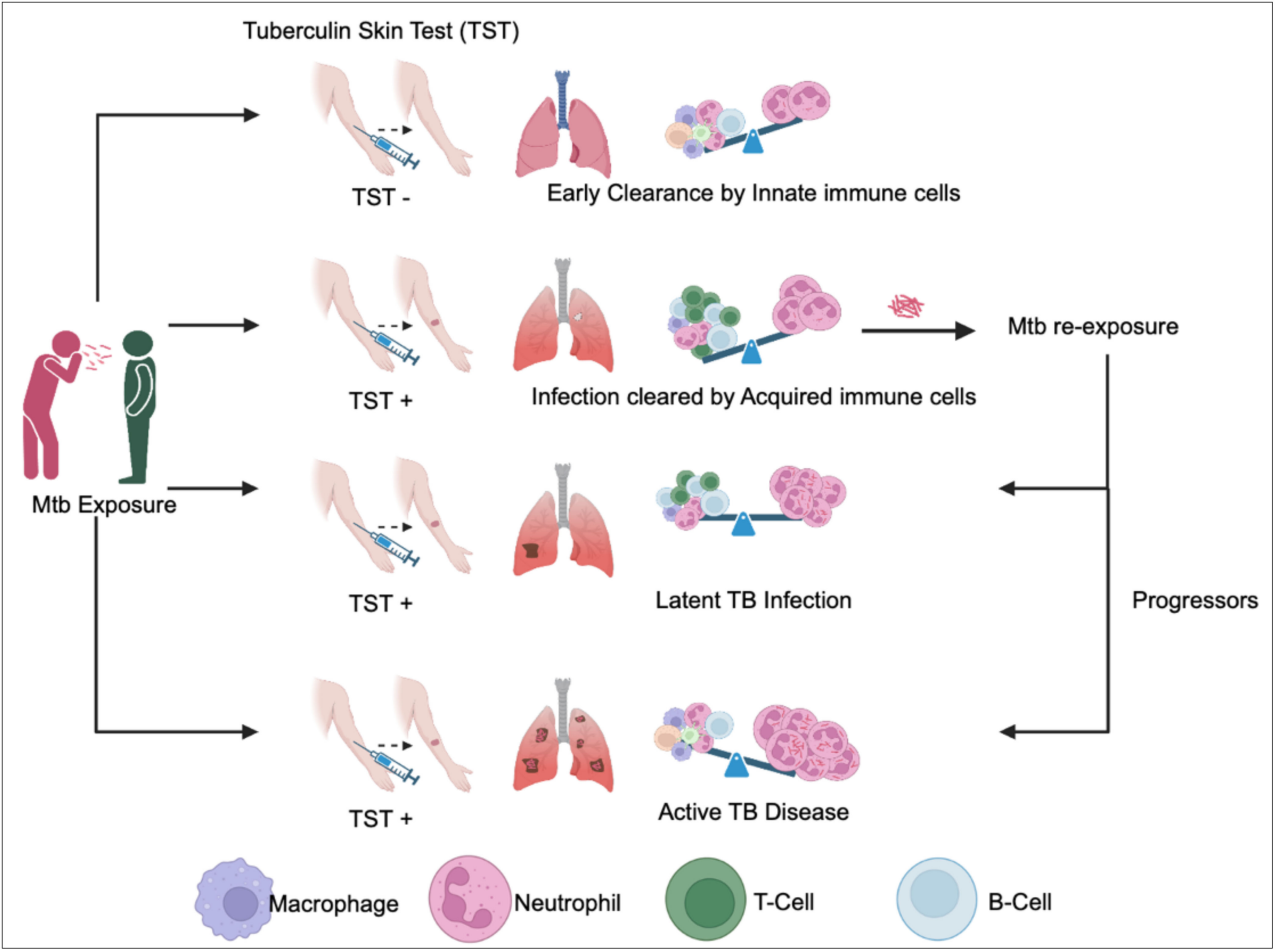
Outcomes of Mtb exposure are determined by immune cells in the lungs. *Mycobacterium tuberculosis* (Mtb) entering through the upper respiratory tract can follow different outcomes. (1) In cases where innate immune cells effectively eliminate Mtb before antigen presentation and T-cell activation, the Tuberculin Skin Test (TST) result is negative (TST^-ve^). (2) Conversely, when innate immune cells fail to eradicate Mtb, T-cell activation occurs in the lymph nodes, leading to the eventual clearance of Mtb by Mtb-specific T-cells. In this scenario, individuals develop Mtb-specific antigens, resulting in a positive TST result (TST^+ve^). (3) If acquired immunity proves insufficient to clear the bacteria, Mtb is contained and enters a dormant state, leading to latent tuberculosis infection (LTBI). Individuals with LTBI possess Mtb-specific T-cell immunity and continue to test positive on the TST (TST +ve). (4) Upon re-exposure to Mtb, the outcome can vary, with some individuals maintaining latent TB infection, while others progress to active TB disease, depending on their immune status. Progressors exhibit heavy neutrophilic inflammation and tissue necrosis. These individuals present at clinics with TST^+ve^ results and manifest TB symptoms. The crucial determinant in controlling or progressing from Mtb infection to active disease lies in the delicate balance between neutrophil granulocytes and Mtb-specific T-cells.

The role of adaptive immunity in the control of *Mtb* infection is well studied and the protective function of T-and B-lymphocytes in anti-TB immunity is widely appreciated. However, upon exposure to Mtb, it usually takes 4-6 weeks for a human host, and 2-3 weeks in mice “to develop antigen specific T-cell responses” in the lymph nodes. Hence, the non-specific innate immune response plays a pivotal role in protecting the host before the onset of adaptive immunity and even leads to early clearance of bacteria (**Figure 1**). Clinical and epidemiological studies further corroborate this concept of early clearance. It is observed that some household contacts of active TB patients remain tuberculin skin test negative (TST^-ve^) even after two years of follow-up. These TST^-ve^ individuals have gene locus mapped to their chromosome that harbors genes of innate immune response such as cathepsins, formyl peptide receptors. In a rabbit infection model Subbian et al. showed that, the abundance of antimicrobial peptides, cathepsins, formyl peptide receptor transcripts in the lung early during infection predicts outcome of infection (1). Importantly, these innate immune transcripts also predict a protective adaptive immune response, and productive inflammatory response. While most of the vaccine candidates tested in experimental animal models of TB induce T-cell memory responses, the effect of innate immune cells and their role during vaccine mediated protection has not been studied in detail and is a topic of wide research interest. A vast body of literature is available on the role and mechanisms of protection conferred by T-and B-lymphocytes during chronic TB. In this review, we will discuss the role of various cells that participate in the innate immunity to Mtb infection at the pulmonary interface and shape adaptive immunity to protect against chronic TB. We argue that a better understanding of the early host-pathogen interactions could inform on future vaccination approaches and devise therapeutic strategies while aiding in disease biomarker discovery.

## 2 Main barrier cells of the lung in host defense against TB

### 2.1 Alveolar epithelial cell

Alveolar epithelial Cells (AECs) form the epithelial barrier in the lung and are the first cells that interact with any pathogen that tries to enter the airways. Type 1 AECs (AT1) are thin, squamous epithelial cells that make up to 90-95% of the airway barrier and are involved in gas exchange between lungs and blood. In contrast, Type 2 AECs (AT2) are cuboidal epithelial cells that make up 5-10% of the barrier and have more pronounced immunomodulatory functions (2–4). Studies have reported that these AECs play crucial roles in initial host defense by secreting antimicrobial peptides, reactive oxygen species, surfactant proteins, lactoferrin, defensins, interferons and other cytokines in response to a pathogen before immune cells infiltrate the lung (4–6). AECs recognize pathogens by specific pattern recognition receptors present on the apical surface and produce chemotactic cytokines/lipids to recruit immune cells to the lung. Reports have also suggested the involvement of AECs in interacting with macrophages, activating neutrophils and participating in antigen presentation to effector T cells by secretion of several chemokines and cytokines, thus bridging the innate and adaptive arms of immunity (4, 6, 7). AECs thereby play critical roles in mucosal immunity by serving as the first physiological and immunological barriers of the lung.

Since AECs are first barrier cells to interact with any foreign pathogen, disintegration of the epithelial barrier impairs the anti-microbial immunity. Disruption of epithelial barrier is seen during respiratory viral infections like influenza, RSV and rhinoviruses resulting in impaired anti-viral defense and altered lung microenvironments. This airway dysfunction potentially leads to secondary bacterial infections promoting bacterial colonization, stimulating hyperinflammatory responses in diseases such as asthma and COPD by secreting proinflammatory cytokines/effectors (7–10).

During TB, AECs have been reported to play important roles in both bacterial dissemination and anti-mycobacterial host defense. Studies show that Mtb are recognized by pathogen recognition receptors on AECs and invade into these non-professional phagocytes. This is thought to help the bacterium escape professional phagocytes like macrophages and prevent subsequent T cell activation. Mtb interaction with AECs induces NF-kB and MAPK pathways through downstream TLR signaling (5, 11). Transcriptome studies of Mtb in A549 cells have revealed a highly metabolic, replication active environment (12). Hence, the AECs act as a replication-conducive reservoir for bacterial growth and helping Mtb crossing the mucosal barrier to enter the circulation for dissemination. The Mtb replication rate is highly enhanced in both AT1 and AT2 cells (3, 12–15). Uncontrolled replication of Mtb within these cells causes cell necrosis releasing the highly virulent bacteria, stimulating phagocytosis, and infection of bystander surrounding cells (13). Mtb has been shown to promote its internalization into these nonprofessional barrier cells by inducing the aggregation of lipid rafts (13, 16). Further, several reports have suggested that Mtb replicating inside the nonprofessional AECs develop increased invasiveness for survival and replication in other professional phagocytes.

AECs are also known to be vital for host defenses against Mtb. They serve as a physical barrier and the first line of structural defense. Additionally, they also secrete a wide array of anti-microbial substances including anti-microbial peptides (e.g., lysozymes, defensins like human β defensin-2 (5, 17, 18), cathelicidin), surfactant proteins (SP-A, B, C and D), proinflammatory cytokines, chemokines, reactive superoxides, all of which collectively help contain Mtb survival (5, 6). An elegant study employing the lung-on-chip infection model have revealed the indispensable need for surfactants in controlling Mtb infection (19). SP-A secreted by AEC has been reported in aiding macrophage phagocytosis of Mtb and SP-D, a carbohydrate binding lectin, has been reported to agglutinate Mtb and inhibit its uptake and growth in macrophages (13, 20). AECs are also known to secrete essential proinflammatory cytokines in anti-Mtb defense including IFNγ, TNFα, GM-CSF. which mediate the recruitment of macrophages and T-cells (6). Additionally, the composition of alveolar lining fluid has been shown to affect the survival and growth of Mtb in AECs (21). Alveolar lining fluid contains hydrolases that alter the cell wall composition in Mtb rendering them susceptible to macrophage killing (22).

AT2 cells are also reported to recruit and interact with innate immune cells, including macrophages, neutrophils, dendritic cells, and adaptive immune cells, e.g., B-cells, CD4+, CD8+ and memory T-cells during TB disease (5, 23, 24). SP-A, SP-D, and matrix metallopeptidase-9 are reported to facilitate macrophage recruitment and phagocytosis. SP-A, CCL-2, TNF and GM-CSF are released from AT2s regulate macrophage cytokine signaling and ROS production. AECs secrete CXCL5, IL-6, IL-8, a chemoattractant via activated TLR2 signaling promotes host defense by recruiting neutrophils from circulation (2, 5). AECs are also reported to interact with neutrophils via ICAM-1 and mediate their activation, promoting neutrophilic inflammation and bacterial clearance (13). AEC also express MHC-I and MHC-II on their surface which enables them to efficiently present antigenic peptides to effector CD4 and CD8 T-cells (6, 25). Numerous studies have shown the role of AEC in influencing T-cell responses. These cells are also reported to express CD1d and MR1, hence can present lipid antigens to iNKT cells and peptides to mucosal Invariant T- Cells (MAIT) to induce IFN-γ production, respectively. AECs effectively activate memory T-cells and induce rapid cytokine signaling following re-infection with Mtb (2, 13, 25–27).

AT2-specific GM-CSF expression is critical for early immunity to Mtb infection, though at later stages GM-CSF is produced by both myeloid and lymphoid cells (6, 24, 28, 29). Interestingly, in a *Legionella pneumophila* infection model, AT2 cells secrete GM-CSF which metabolically reprogram monocytes through in a GM-CSF receptor dependent (Csf2r) manner to augment antimicrobial function of these cells (30). However, the crosstalk between AT2 cells and other lung cells, especially with resident/recruited immune cells in the protective immunity against TB is not fully understood.

*In-vitro* studies using A549 cells and *in-vivo* studies in mice have reported the dependence of Mtb on RD-1 locus and more precisely, Esta6 in necrosis, cytolysis, and pulmonary dissemination of the bacteria across physiological epithelial barriers (2, 3, 31). Though reports are still emerging, AECs clearly have an important role in the host response to TB and future investigations on their role in immunity to TB may reveal novel therapeutic avenues and inform vaccination approaches.

### 2.2 Endothelial cell

Endothelial cells are flat, squamous cells that line the vasculature and the lymphatic vessels (blood EC and Lymphatic EC respectively) and serve as the first responders to any foreign material entering the blood or lymph nodes. They form a semi-permeable layer and aid in the transport of cells across the barrier, especially during inflammation when immune cells are recruited, and they enter the interstitium through the endothelial barrier (32, 33). ECs line the pulmonary vasculature and facilitate in gas exchange. ECs regulate metabolic homeostasis, vascular permeability, and hemodynamics under basal physiological conditions. These cells are among the first responders to infection and act as conditional immune cells (32, 34). During inflammatory conditions, ECs facilitate immune cell transport by trans endothelial migration (TEM), recognize pathogens by increasing expression of PAMP receptors (TLRs, NLRs) on cell surface (32, 34), present antigens to T-cells and iNKT cells (32, 34, 35). They have also been reported to be antimicrobial and restrict the growth of certain bacteria (*S.aureus)*, parasites and viruses but can also harbor the growth of certain intracellular bacteria like Mtb. ECs produce reactive oxygen (ROS), nitrogen intermediates (RNIs) and antimicrobial peptides like defensins to control bacterial growth (32, 33).

Endothelial dysfunction underlies the pathogenesis of various lung and cardiovascular diseases. TEM of immune cells across the endothelial monolayer is vital in case of COPD and asthma (36). Increased serum levels of leukocyte adhesion molecule ICAM-1 have been reported in COPD patients, which mediates emphysema, increased inflammation, and reduced lung function (37, 38). Similar increase in adhesion molecules has been reported in asthma patients, corresponding to increased TEM, influx of inflammatory immune cells and increased cytokine production by EC’s leading to lung damage (36, 37, 39). EC production of cytokines and chemokines play a definitive role in the pathogenesis of influenza infection by production of proinflammatory cytokines, chemokines and recruiting early innate immune cells thereby regulating the cytokine storm seen during influenza (40, 41).

During TB, both vascular and lymphatic ECs succumb to Mtb infection and can help in dissemination of bacteria through the bloodstream and lymph respectively, as evidenced by their ability to be internalized by human umbilical vein endothelial cells (42), various endothelial cell lines, and lymphatic endothelial cells cultured from TB patients (35, 43, 44). Studies have shown that bacterial uptake into EC are mediated by cytoskeleton and their endocytosis requires Rab5 and Rab7 (45). ECs have been proposed to be a niche for bacterial replication which requires the virulence locus RD1 (34, 35). The vascular endothelium serves as a passage for infiltrating immune cells to the infected sites to facilitate early inflammatory response to the pathogen leading to the formation of granulomas (46). Nascent granulomas are extremely vascularized though fibroblasts and calcified deposits are often observed as these inflammatory structures mature. Recent studies have revealed that Vascular Endothelial Growth Factor (VEGF) levels are increased in the serum of TB patients (47–49). VEGF is essential for angiogenesis and endothelial cell formation. Mtb infected macrophages secrete VEGF, that requires RD-1 dependent virulence factors (50). This further promotes the recruitment of endothelial progenitors from the bone marrow and the formation of new blood vessels, essential for the extrapulmonary dissemination of the bacteria (34, 50, 51). VEGF inhibition reportedly reduces bacterial dissemination to lung draining lymph nodes and spleen (52). Potential anti-VEGF treatments are being investigated to improve TB pathophysiology (46, 53). Mtb is found in lung and splenic ECs in patients with extrapulmonary TB, which emphasizes the role of ECs in dissemination of the bacteria to extrapulmonary sites (34, 54). Extracellular vesicles from Mtb*-*infected macrophages that contain bacterial virulence mediators activates TNF α, NF-kB and Type I IFN pathways in ECs, critical for host defense (51, 55). Like angiogenesis, Mtb stimulates lymph angiogenesis, the production of new lymphatic vessels which might serve as a basis for lymphatic transmission of the pathogen and subsequently establishing extrapulmonary TB (52). Recently, it was reported that Mtb forms cords indicating thousands of bacteria engaged end to end in human lymphatic ECs. Cording of Mtb is linked to increased virulence and immune evasion (55).

Mtb infects both vascular and lymphatic ECs (43, 54). Expression of PAMPs like phthiocerol dimycocerosates (PDIM) and mannosylated lipoarabinomannan (Man-LAM), on the cell surface of the bacterium facilitates recognition by mannose receptors and infection of ECs (34, 35, 43). Recent studies revealed that PDIM-deficient Mtb could not survive in human ECs due to the inability to disrupt the phagosome membrane, a key attribute for intracellular replication for Mtb (44). Moreover, Mtb inhibits endosomal maturation to survive intracellularly. A fraction of the bacteria exploits the autophagy pathway to enter the autophagosomes and replicate within ECs (34, 43).

Although ECs have been studied as being able to internalize Mtb *in-vitro*, the replication dynamics within vascular and lymphatic ECs *in vivo* remain obscure. Further studies are needed to form a mechanistic basis of how the bacteria gain access to the blood and lymph, disseminate from ECs to other surrounding cells and spatio-temporal dynamics of ECs in granuloma, their crosstalk with immune cells will contribute to our understanding of the pathogenesis of TB.

## 3 Major immune cells in Innate host defense against TB

### 3.1 Macrophages

Macrophages are phagocytic, tissue resident myeloid cells that constitute the first-line immune sentinels. In the lungs, based on the location and surface marker expression, macrophages can broadly be divided into alveolar macrophages (AM) that are present in the airways and interstitial macrophages (IM) that are in the lung parenchyma. AMs, due to their positioning in the airways have essential roles in maintaining barrier integrity, lung epithelial lubrication by regulating surfactant and immunoregulatory cytokine production, removal of dead cells by efferocytosis during homeostatic conditions. IMs in the lungs, regulate T-cells and DCs by producing IL-10 during infection (56–59). Based on their mode of activation, macrophages are classified as M1 (Classically activated by Th1 cytokines) or M2 (alternatively activated by Th2 cytokines). Their abundance in the lung milieu impacts the response to infection (56, 60, 61).

Alveolar macrophages are lung resident, terminally differentiated cells, that accumulate in the lungs during fetal development and are maintained by GM-CSF and TGF-β production by AECs (62). Following infection, they are continually replaced by monocyte derived macrophages. AMs are the primary responders to infection as they constantly screen pathogens and defend the lungs. Their phagocytic properties are exploited by several pathogens and macrophages are hence involved in the immunopathology of numerous lung infections, including influenza, respiratory syncytial virus, COPD, cystic fibrosis, asthma, COVID-19, and TB (56, 63–66).. During infection, there is an increased recruitment of blood monocytes to the lung, where they differentiate to macrophages. Activated monocyte derived macrophages (MDMs) are potent source of proinflammatory cytokines, reactive oxygen/nitrogen species, aggravating the inflammation. Additionally, macrophages can interact with neighboring AECs, other immune cells like DCs, neutrophils, monocytes, and T cells to regulate the inflammatory microenvironment of the lung (56, 60, 61, 67). Recent research has shown how macrophages proliferate and secrete cytokines more vigorously upon restimulation (67–69).

Classically, AMs are the immune cells that have been associated with TB immune response. Mtb enters the airways through the upper respiratory tract and is taken up by the airway resident AMs. The bacterium replicates within these phagocytes and travels to the lung parenchyma where it disseminates into other immune/stromal cells (70–76). AMs initially upregulate their defenses, especially NRF2 mediated antioxidant pathways in the early stages of infection but eventually the bacterium subvert the antimicrobial response of these cells (77). While the mechanisms underlying Mtb hijack of AMs is still a subject of extensive research, it is well established that the bacterium manipulates phagosome maturation by preventing phagolysosome formation in AMs in an ESX-1 dependent manner to prolong its survival in these cells (70, 73, 78). Mtb infection regulates macrophage autophagy, apoptosis, inflammatory cytokine production, inflammasome formation, antigen presentation, immune-cell crosstalk. Mtb shuts down the host favorable apoptosis and induces necroptosis via TNF and type I IFN secretion (79–83). Ironically, macrophage death leads to increased Mtb growth and replication (72). Mtb aggregates can feed on the dead macrophages in an ESX-1 and bacterial lipid phthiocerol dimycocerosate (PDIM) dependent manner, without the need for live AMs, thereby avoiding phagocytosis (84).They can further kill other macrophages resulting in a cascade of cell death and inflammation, boosting Mtb transmission. AMs infected with Mtb drive the formation of granulomas, along with other macrophages like interstitial and foamy macrophages, epithelial cells and other immune cells like neutrophils, monocytes, DC, NK cells, T-cells, B-cells. While granulomas have been traditionally believed to contain infection, emerging evidence posit a pathogenic function for these inflammatory structures (85–87). Following infection, emergency myelopoiesis is initiated in the bone marrow, which leads to the recruitment of monocyte derived macrophages (recruited IMs) to the lungs, which are short lived, glycolysis driven and can effectively contain bacterial growth. *In-vivo* studies in mouse models of TB have shown that AMs are more susceptible to infection as they present less oxidative stress phenotype and higher replication rates in comparison to IMs, which have better restrictive capabilities. These two types of macrophages also have altered immunometabolism with permissive AMs directed towards “increased fatty acid metabolism” and IMs relying on glycolysis (74, 75).. Mtb drives an M2 like anti-inflammatory phenotype in AMs to replicate within these cells whereas IMs have a more replication limiting M1 like pro-inflammatory phenotype (71, 76, 88).

Apart from these, Mtb initiate the fusion of macrophages to form multinucleated giant cells that have reduced phagocytic capability and can serve as a permissive niche for bacterial survival and propagation (76, 85, 89). Mtb also initiate the formation of fatty-acid rich foamy macrophages to serve as nutrient rich reservoirs for extended periods of time, during dormancy. These foamy macrophages are characteristics of TB granulomas and significantly harbor replicating bacteria (90–92). Human AMs have the antimicrobial capacity to effectively kill Mtb, but the bacterium invokes a change in the transcriptome of macrophages towards a lipid accumulating, proinflammatory phenotype thereby perturbing their antibacterial defenses as evidenced by transcriptome studies from AMs isolated from the bronchoalveolar lavage of human TB patients, and comparing them with the healthy volunteers (93). *In-vivo* studies with mouse models of TB disease have shown similar dependency of Mtb on fatty acid metabolism (92, 94).

Elegant studies using Bacillus Calmette-Guérin (BCG) vaccinated mice have revealed the importance of macrophages in innate immune memory. BCG reprograms the hematopoietic stem cells in the bone marrow. These changes render monocyte progenitors to generate macrophages that are more proinflammatory and restrict virulent Mtb replication. However, this phenomenon is drastically opposite in the case of virulent Mtb infection, where myelopoiesis was restricted and the Mtb trained macrophages could not efficiently control infection (68, 69). Future studies in the areas of how Mtb evade the antimicrobial immunity exerted by macrophages, and a detailed mechanistic insight into macrophage functions in trained immunity may help in designing next generation TB vaccines.

### 3.2 Neutrophils

Neutrophils are the most abundant white blood cells in the human body and play crucial roles in protective immunity against a variety of pathogens. Recently, both experimental and clinical studies of active pulmonary TB have shown that this disease is accompanied by massive influx of neutrophils into the lung tissues (95). In humans, active TB and disease severity have been associated with neutrophilic response (96). An increase in neutrophils, and high neutrophil/lymphocyte ratio, distinguished TB patients from tuberculin skin test-positive healthy contacts. Within this group of TB patients, an extensive neutrophilic response is a sign of TB severity and specifically has been associated with damage to the pulmonary architecture (97). Neutrophils are the dominant cell types in tissue biopsies obtained from pulmonary TB patients that harbor Mtb (98). During pleural TB, the accumulation of neutrophils in pleural effusions was associated with significantly higher inflammatory serum markers. Frequent detection of Mtb in the pleural fluid is associated with poor prognosis.

Although the specific role and clinical significance of neutrophils in tuberculosis are still controversial, these studies established a close correlation between the development of tuberculosis and infiltration of the tissues by neutrophils. However, the role of circulating neutrophils in the development of tuberculosis is largely unknown. Peripheral blood neutrophil-specific type I IFN-inducible genes have been identified as the unique transcript signature of active TB that accurately predicts patients with active disease from LTBI (99). Very recently, the same group has identified a cross-species signature specific to neutrophils that distinguishes hosts likely to progress to active disease from those that contain the infection. Another independent study recently showed that the presence of neutrophils and neutrophil granule protein S100A9 in the TB lesions of both nonhuman primates and humans likely predicts the latent infected and progressors to active disease (100, 101). Altogether, these reports establish neutrophils as the pathologic mediator of TB disease.

#### 3.2.1 Antimicrobial function of neutrophils during TB

Neutrophils are the first phagocytes recruited from the vasculature to the pulmonary interstitium during TB. Upon exposure to Mtb, neutrophil blood counts in human pulmonary TB (PTB) contacts are initially higher than in unexposed control subjects. Individuals in contact with pulmonary TB patients are less likely to be infected with Mtb if they have higher peripheral blood neutrophil counts. In one study, it was shown that one hour after *in vitro* infection with virulent Mtb and stimulation with TNF, neutrophils suppressed the growth of the bacteria by 50–95%, although significant variability in mycobactericidal capacity between donor neutrophils was reported (102). Neutrophils are the early phagocytic cells in TB granulomas and kill Mtb by causing oxidative damage (103). Neutrophil-depleted whole blood had a decreased capacity to control Mtb infection *ex vivo*. Taken together, these results indicate that the mycobactericidal function of neutrophils might be key to innate immune control of Mtb.

#### 3.2.2 Pathological role of neutrophils during TB

Protective immunity to TB involves immune responses within the pulmonary airways, which can lead to exacerbated inflammation and immune pathology. Neutrophils, though increasingly linked to the development of inflammatory disorders, have been less well studied in relation to TB-induced lung pathology. Neutrophils’ mode of action and their specialized functions can be directly linked to TB-specific lung tissue damage observed on patient chest X-rays at diagnosis and contribute to long-term pulmonary sequelae. It highlights the significance of neutrophil function on TB disease outcome and underlines the necessity of monitoring neutrophil function for a better assessment of the immune response and severity of lung pathology associated with TB.

Infection in highly susceptible strains of mice shows the detrimental effect of unchecked neutrophil recruitment on infection control and eventually an increase in TB disease severity. TB lesions of various susceptible mouse strains contain a substantial number of necrotic neutrophils, whereas more “resistant” mouse strains develop lesions with only scattered neutrophils and little or no necrosis (104). In humans, as in the mouse model, necrotic neutrophils are unable to control Mtb infection (105). Phagocytosis of Mtb-induced necrotic neutrophils by macrophages promotes bacterial growth. These findings indicate that persistent neutrophil influx to the infected lung might give rise to necrotic inflammation and impaired bacterial control. By using high-throughput bacterial genetics and cell sorting-based approaches, we were able to demonstrate that neutrophil-rich TB lesions serve as pathogen-permissive sites, and these cells serve as the host niche for bacterial replication and promote tissue-damaging inflammation. We also demonstrated that a Ly6G^lo^ population of neutrophils accumulates in the murine lung during TB and positively correlates with disease severity. Importantly, these neutrophil populations are relatively long-lived compared to conventional neutrophils and, owing to their impaired ability to express antimicrobial proteins, serve as a permissive cellular niche for Mtb in TB lesions.

Neutrophils remained understudied in TB, partly due to their low abundance in the most commonly used murine models, namely C57BL/6 and BALB/c mice, which are relatively resistant to Mtb infection. In a classic low-dose aerosol infection of Mtb, neutrophils have been found only within the first 14 to 21 days post-infection in the lungs but are barely detected from lung lesions at later time points. These lesions consist of inflammatory infiltrates dominated by macrophages, T- and B-lymphocytes but do not represent necrotic granulomas as seen in human patients. Moreover, depletion of neutrophils in these mice did not change disease outcome. The decreased abundance of neutrophils from these lesions was due to the expression of IFNγ mediated Nos2 expression after the onset of adaptive immunity that restrains an unchecked IL-1 mediated neutrophil influx. Mice lacking Nos2, IFNγ or T-/B-lymphocytes succumb to disease by developing neutrophil-rich TB lesions, high bacterial burden (106). Indeed, depletion of neutrophils from these susceptible mice ameliorates disease. Neutrophils represent a significant proportion of the cellular infiltrates at the site of infection of mouse strains that are highly susceptible to TB, e.g., C3HeB/FeJ and I/St mice (107–109). In these strains, neutrophils were associated with necrotic tissue damage and early mortality of the animals. Neutrophil depletion in Mtb-infected I/St mice reversed wasting disease, reduced mycobacterial burden, pathology, and improved survival. C3HeB/FeJ mice are hyper susceptible to Mtb infection compared to other inbred mouse strains. Mtb infection in these mice elicits lung pathology with structured granulomas and neutrophil-driven, centrally caseating, necrotizing, and sometimes even cavitary lung lesions like those in humans. Although the mechanisms behind early mortality of animals after infection are still elusive, genetic predisposition plays an important role. Pan et al. identified an allele in C3HeB/FeJ mice, called the “super susceptibility to tuberculosis 1” (sst1) locus. Animals inheriting the susceptible version of the sst1 allele (sst1^s^) develop necrotic lesions, fail to control the growth of virulent Mtb, and ultimately exhibit reduced survival rates. Importantly, these lesions contain clusters of neutrophils similar to human TB granuloma. Recent characterization of the genes in the sst1 locus identified a single gene, Sp140, which is postulated to negatively regulate type I IFN production that is pathogenic during TB (110, 111). Furthermore, type I IFN production during TB regulates neutrophil influx to the lung and activates neutrophils to produce neutrophil extracellular traps (NETs) (112). Indeed, heterogenous population of neutrophils based on their buoyant density, have been reported in TB. It is proposed the low-density neutrophil subsets are highly pathogenic owing to their ability to form NETs compared to the conventional mature neutrophils (113). While concrete evidence for neutrophil subsets in TB is lacking, these studies have set the foundation into this exciting new area of neutrophil biology and implications in TB pathogenesis.

Persistent influx of neutrophils into TB granulomas in mice is causal to failed immune control. Moreover, there is a growing appreciation for the role of neutrophils in active TB in humans and high neutrophil counts associate with poor prognosis (114). Neutrophil depletion studies in animal models of TB have suggested a dichotomous role of neutrophils in TB as these cells are antimicrobial early and pro-bacterial during the chronic phase of infection. Interestingly, neutrophils affect granuloma outcomes in non-human primates, as depleting neutrophils from granulomas with high bacterial burden reduces bacterial replication in those granulomas, while depleting neutrophils from granulomas with low load increased the number of bacteria per granuloma (108). These results suggest that the degree to which neutrophils contribute to pathogenesis vary along a spectrum at the individual granuloma level. Thus, the role of neutrophils in the immune response to Mtb particularly studying their functions at different stages of the disease may provide a deeper understanding of TB pathogenesis. Overall, a better understanding of the role of neutrophils in TB can provide insights into disease progression, immune response, and potential therapeutic interventions.

### 3.3 Eosinophils

In acute bacterial infections and tuberculosis, neutrophils are often the first granulocytes to respond, playing a critical role in early immunity (115). Traditionally it has been believed that neutrophils are the first immune cells to recruit to Mtb-infected lung (116). However, this paradigm has been recently challenged by elegant studies in rhesus macaques and mice, demonstrating that eosinophils are the first granulocytes to be recruited to the airways (117). Eosinophils are involved in a wide range of immune-mediated responses, including those to allergens, parasites, infections associated with type-2 immunity, fibrosis, wound healing, tissue repair, and remodeling. They possess large cytoplasmic granules that store pre-formed bioactive molecules such as cytokines, chemokines, lipid mediators, and cationic granule proteins, allowing them to tailor their responses to different stimuli (118).

Eosinophils can be distinguished from neutrophils by the intracellular expression of eosinophil peroxidase (EPX). Eosinophil recruitment to the airways occurs as early as one week after Mtb exposure, surpassing neutrophil numbers by two weeks. Similar enrichment of eosinophils, not neutrophils, has been observed in the lung vasculature of infected mice, with recruitment into the lung parenchyma starting as early as one week after infection. Interestingly, this early eosinophil recruitment is not dependent on the classical C-C Motif Chemokine Receptor 3 (CCR3) pathway typically associated with eosinophil responses. Instead, the eosinophil-intrinsic expression of G Protein-Coupled Receptor 183 (GPR183), an oxysterol-sensing chemotactic receptor not previously considered for eosinophil migration is required for their recruitment. Oxysterols, which are bioactive cholesterol derivatives, are synthesized by the enzymatic action of cholesterol-25-hydroxylase (Ch25h). This enzyme is regulated by type I interferons (IFN) and Toll-Like receptor (TLR) activation, implicated in TB pathogenesis. The recruitment and migration of eosinophils into Mtb-infected lungs is dependent on Ch25h-derived oxysterols, as evidenced by the abrogation of eosinophil enrichment and migration in Ch25h-deficient mice. Importantly, Mtb infected alveolar macrophages, but not uninfected macrophages, expressed higher levels of Ch25h early after Mtb infection. This suggests that infected alveolar macrophages increase Ch25h expression and oxysterol production to selectively recruit eosinophils for cell-cell interactions. Direct cell-cell interactions between eosinophils and Mtb-infected alveolar macrophages have been demonstrated in a dynamic imaging model using Mtb-infected lung explants (119). Whether eosinophils interact with infected macrophages in an oxysterol dependent fashion or other non-immune cells in the mucosal barrier initiate this cascade remains to be explored. Nevertheless, the emergence of these type 2 innate immune cells as the first responders to Mtb infection opens a completely new area of investigation. Moreover, eosinophils have been proposed to play a protective role during TB as eosinophil deficient mice develop immunopathology and succumb to infection. Future investigation into the role of these cells in Mtb resistance and disease tolerance to maintain tissue homeostasis may reveal insights into TB protection and pathogenesis.

### 3.4 Inflammatory monocytes

Monocytes are part of the mononuclear phagocytic system and are recruited from the myeloid compartment following infection. They can differentiate into monocyte-derived macrophages or monocyte-derived dendritic cells and play a vital role in innate immunity during various infections. Circulating monocytes can either be Ly6C^hi^ ‘inflammatory’ monocytes or Ly6C^lo^ ‘patrolling’ monocytes. Following infection, monocytes are rapidly recruited from the blood to differentiate into effector cells, making them pivotal for innate immune defense. In both mice and humans, monocytes can be classified into classic, intermediate, or non-classical subsets based on their surface marker expression, each with diverse functions. While the classic monocytes are the primary phagocytes, intermediate monocytes produce reactive oxygen species (ROS), present antigens to CD4+ T-cells and implicated in lymphocyte activation. Non-classical monocytes posess pro-inflammatory roles (120–122).

In response to inflammatory signals during infection, Ly6C^hi^ CCR2^+^ monocytes expand in the bone marrow and are recruited to sites of infection. These monocytes are referred to as “inflammatory monocytes” and have a hyperinflammatory phenotype, contributing to immunopathology in several lung infections (120, 123–125). In influenza infections, CCR2^+^ monocytes are recruited to the lungs and contribute to overwhelming inflammatory responses, leading to worsened disease (126, 127). Studies have also shown that CCR2^+^ inflammatory monocytes drive lung injury and mortality in juvenile mice during influenza infection, highlighting the detrimental effects of this cell type (128). Additionally, these cells with a bias towards hyperinflammatory responses have been implicated in the immunopathology of other respiratory diseases such as asthma, COPD, cystic fibrosis (122, 123, 129).

In the context of tuberculosis, human patient research suggests that the levels of intermediate and non-classical subsets (CD16^+^) monocytes are elevated during TB disease (130–132). Interestingly, their frequency reduces post anti TB treatment (133, 134). High monocyte to lymphocyte ratio has been considered as a biomarker for active TB disease (135, 136). CD16^-^ classic monocytes are considered to have anti-mycobacterial function, and these subsets are diminished following TB infection (137). Chemokine ligands for CCR2 (CCL2/MCP-1, CCL-7, CCL8) secreted by mononuclear cells infected by Mtb are necessary for the recruitment of CCR2^+^ monocytes from the bone marrow via blood. These inflammatory monocytes can differentiate into macrophages (phagocytic) or dendritic cells (antigen presentation) and carry out a range of effector functions, including the production of inflammatory cytokines (TNF-α, IL-1β, ROS) (138).

Studies report that CD16^+^ monocytes promote intracellular bacterial survival and affect their capacity to differentiate into antigen-presenting monocyte-derived dendritic cells, whereas CD16^-^ monocytes are more anti-bacterial (139). Furthermore, it has been reported that Mtb can drive CD16^-^ monocytes towards an anti-inflammatory M2 macrophage phenotype, increasing the permissibility of mononuclear cells (131).

CCR2 expression on inflammatory monocytes is essential for their recruitment to the lungs. Studies with *Ccr2^-/-^* mice have suggested increased susceptibility to TB disease in the knockout mice. Marked differences in bacterial burden, monocyte recruitment, antigen presentation, and T-cell priming were observed following Mtb infection in wild-type and *Ccr2^-/-^* mice, reinforcing the role of CCR2 in the recruitment of inflammatory monocytes and its protective function during TB disease (140). These CCR2^+^ inflammatory monocytes have also been shown to mediate Mtb specific CD4 T-cell responses. This study further reported that monocyte-derived dendritic cells, rather than inflammatory monocytes are responsible for T-cell priming in pulmonary lymph nodes (141).

Recent research on trained immunity by BCG in Mtb infections has revealed that monocytes/macrophages derived from the hematopoietic stem cells (HSC) in the bone marrow are educated by BCG. These circulatory monocytes, when recruited to the site of infection, exhibit high protective properties. Notably, studies using parabiosis have shown the protective role of CCR2+ circulatory monocytes, trained by BCG, in reducing bacterial burden and lung damage in *Ccr2^-/-^* mice during TB disease (68). Furthermore, it has been shown that this reprogramming of HSC and training of monocytes/macrophages by BCG are starkly opposite to the changes caused by Mtb infection. TB infection leads to HSC exhaustion, diminished control by bone marrow-derived monocytes/macrophages, and specifically, a reduction in the egress of CCR2+ monocytes into the blood (69).

While IFNγ is vital for host defense against Mtb, Type I IFN (IFN-I) signaling promotes infection. Interestingly, during chronic inflammation, IFN-I signaling promotes the production of CCL-2, CCL-7, CCL-8, enhancing the recruitment of Ly6C+ CCR2+ inflammatory monocytes. During TB, IFN-I signaling-mediated recruitment of inflammatory monocytes augments the intracellular permissibility of monocytes and promotes their differentiation into an Mtb-tolerant M2 macrophage cell type with a non-inflammatory phenotype, while reducing monocyte differentiation into dendritic cells and impairing antigen presentation and T-cell priming (142).

While the protective and disease-promoting roles of inflammatory monocytes have been studied, the factors that mediate their differentiation into pathogen-permissive macrophages or antigen-presenting dendritic cells are not well understood. Further studies are needed to exploit the protective functions of CCR2+ monocytes against TB pathogenesis.

### 3.5 Dendritic cell

DCs are best known as professional antigen presenting cells (APC), that present peptide antigens processed via endocytic pathway and loaded onto MHC-II molecule to effector CD4 T-cells. They are vital bridges between the innate and adaptive arms due to antigen presentation. They act as essential cell types for front line defense against pathogens. DCs are present throughout the lung tissue beneath the epithelial layer and constantly sample antigens in the airways. DCs are a heterogenous population and include subtypes- Conventional/Myeloid DC (cDCs), plasmacytoid DC (pDCs) and monocyte derived DC (moDCs). Myeloid DCs sample and recognize PAMPs and DAMPs in the airways and lungs. They process and present these antigens to naïve T cells in the draining lymph node and are pivotal in initiating T cell responses. DC are conventionally present in an immature state in the lung. Upon encountering antigens, they become mature migratory cells that travel to the lymph nodes. Similarly, due to their constant sampling of antigens, DCs can also process ‘self’ antigens and migrate to the lymph nodes as immature cells and induce immunogenic tolerance towards these antigens. Additionally, DC also have important roles in mediating B cell responses, both directly and indirectly via CD4 T cell stimulation. DCs are essential in maintaining the resident memory T cell pool. Plasmacytoid DCs are potent producers of IFNγ and are essential for antiviral defense. Following infection, blood monocyte derived DCs are rapidly recruited to the lungs. Apart from activating the adaptive arm, DCs are also potent sources of proinflammatory cytokines and can modulate the inflammatory lung environment (143–148).

During influenza infection, inflammatory DCs are rapidly recruited to the lung in a CCL2 regulated manner. DCs are essential for orchestrating innate immune responses by early recognition of viruses and recruitment of NK cells (149). Interestingly, due to their ability to ingest and transport antigens to lymph nodes, DCs mediate the pathogenesis of various airway inflammatory diseases, e.g., asthma and COPD (150). Different subsets of DCs are involved in activating T-cell immunity, causing anergy and mediating inflammation is a subject of wide research interest (143, 144, 146).

Mtb is recognized by DCs via TLR9, and this initiates the uptake of the bacteria (151, 152). Since Mtb is an intracellular pathogen, it is processed via cytosolic pathway and antigens loaded onto MHC-1 are presented to cytotoxic CD8 T-cells. This enhances cytokine secretion by T-cells and recruitment of other immune cells like neutrophils and monocytes facilitating bacterial clearance (152). Both classical and monocyte derived DCs are important for CD4 T-cell priming during Mtb infection. Mice that are depleted in DCs have compromised CD4 T cell response, increased bacterial load and decreased survival (153). Recent reports have suggested Mtb antigens drive DC assisted activation and proliferation of regulatory T cells in the lymph node (151). This is postulated to delay the activation of effector T cells and ultimately increase the lung bacterial burden. *In-vitro* studies in human DCs have shown that Mtb suppresses proinflammatory cytokine secretion and integrin expression on DCs, impeding their ability to attach to endothelial monolayers which might support the delay in initiating adaptive T cell responses (154).

DCs are one of the first leukocytes to be infected with Mtb in the lung. Following infection, immature DCs (iDC) upregulate MHC-II and CD40 expression. These iDCs travel to the mediastinal lymph nodes to prime T-cells to generate Mtb specific effector CD4^+^ and CD8^+^ T-cells to initiate adaptive immunity. These effector lymphocytes travel back to the lung and mediate bacterial clearance. DC are the first cells to initiate protective T-cell responses in the lungs after Mtb infection (153, 155). This pathway is known to be delayed in active TB disease. Recently, a study showed that delivering Mtb primed DC to infected mice or activation of host DC can activate CD4 T-cell responses rapidly paving way towards improvement of DC vaccines (156).

Findings from human TB patients have shown that circulating DC subsets are depleted from the blood compared to healthy donors and are accumulated in infected granulomas (157, 158). This indicates recruitment of DCs to disease sites. These DCs prime T-cells locally as well as move to lymph nodes to present processed antigens to T-cells in a CCR7 dependent manner (159, 160). Granulomas reportedly recruit inflammatory dendritic cells which apart from processing antigens contribute to the cytokine milieu by producing IL-12, TNFα, IL1β, IL1α, IL-6 and IL-10. These cytokines mediate inflammation, T-cell priming, Th17 activation and direct bacterial containment (152, 155, 161).

Using reporter strains of bacteria, studies have uncovered that Mtb rapidly infect DCs *in-vivo* and negatively impact their antigen presentation functions (162, 163). Mtb inhibits the maturation of DCs by restricting the production of IL-12 and other proinflammatory cytokines (164). This results in inefficient transport of DCs to lymph nodes and compromised T-cell priming. Myeloid DCs are one of the major subsets of infected cells in the lung and lymph nodes at the peak of infection. Reports have suggested that the DCs that are infected with Mtb are the least efficient in moving to the draining lymph node and priming specific T-cells. Mtb also interferes with antigen presentation by DCs and infected DCs have less antigens presented on MHC-II. Infected inflammatory DCs that are present in granulomas exhibit a CCR7^low^ phenotype indicating compromised migration and antigen presentation. Interestingly, most DCs in the lymph nodes that effectively induce T-cell response are not Mtb infected, which suggests that Mtb subverts host immunity to establish infection (159). Hence DCs are prime Mtb targets as well as being crucial in wiring host immunity. However, the role of different subsets of DCs especially the type I IFN producing pDCs in TB immunity are poorly understood. An improved understanding of these subsets will pave ways for innovative vaccination approaches against Mtb infection.

### 3.6 Natural killer cells

NK cells are resident lymphocytes in the lung and are known for their quick responses to respiratory infections and constitute about 5-15% of the circulating lymphocytes in the peripheral blood. Since the lung microenvironment is constantly exposed to foreign particles, resident NK cells serve as one of the first lines of pulmonary defense (165–168). These innate cytotoxic effector cells are classified under group 1 Innate Lymphoid cells (ILC) and are early producers of a wide range of effector, immunomodulatory cytokines such as IFNγ, TNFα, GM-CSF and maintain a mature phenotype in the lung. NK cells are recruited to the lung soon after exposure to an infectious agent to release effector cytokines and to facilitate the activation of cytotoxic T cell responses (166, 168–171). Therefore, these cells serve as vital bridges between innate and adaptive immunity.

NK cells are not phagocytic but mediates cytotoxicity of infected cells directly by releasing perforin, granulysin, complement factors to form membrane attack complexes. By producing granzyme B or by expressing FasL on their surface that recognizes the Fas-receptor on infected cells, NK cells induce cell apoptosis (172–174). NK cells activate infected macrophages through NKp46, but the exact functional roles are unclear. Host cells undergo surveillance by NK cells and infected cells are either recognized by pathogen/host derived ligands on their surface or by down-regulation of self-proteins like MHC-1 (non-MHC restricted cytotoxicity) (175). The role of NK cells in defense against cancers, respiratory infections and airway inflammatory diseases caused by various bacterial or viral pathogens is well documented (176, 177). Evidence from influenza virus infections suggest that NK cells are required for antibody dependent cell mediated cellular cytotoxicity (ADCC) to orchestrate viral clearance (166, 169). Increased NK cells and the role of NK cells in recruitment of cytotoxic T-lymphocytes and Th2 cells have been reported in influenza, RSV infections, asthma, COPD; but how their functions are regulated is not well understood (165, 169).

In the case of TB, although there have been conflicting reports from human studies indicating decreased levels of NK cells with disease (178–181) or no significant differences (182, 183), a loss of NK cells and its activation markers is shown to correlate with active disease (184). A recent study shown reduced degranulation and IFNg in TB patients pre-treatment when compared to post-treatment (185). Human peripheral blood NK cells recognize Mtb directly by TLR2 and Nkp44 binding (186, 187). NK cells help in initial containment of bacterial growth by mediating cytotoxicity of infected cells and cytokine signaling in the granuloma (172, 185, 188). Apart from direct interactions with infected cells, NK cells regulate effector functions by IFNγ and TNFα mediated formation of ROS and RNS within macrophages and monocytes. IFNγ is mainly produced by Th1 lymphocytes and essential for inducing an antimycobacterial program in macrophages. IFNγ from NK cells has been shown to be indispensable for the survival in RAG^-/-^ mice post Mtb infection (189). Similarly, NK cell production of IFNγ is critical for immunity to TB indicating their imperative need in immunocompromised patients and in HIV-TB coinfection. Although activated NK cells accumulate rapidly and mediate host defense via perforin, granzymes and IFNg following Mtb challenge, studies in C57BL/6 mice showed no changes in bacterial burden post NK cell depletion that suggests NK cells do not directly regulate TB immunity and other cells could compensate for NK cell mediate defense in an immunocompetent host (172, 190). Cytokines including IL-2 and IL-12 and glutathione, which can balance oxidative stress can activate NK cell function to control TB outcome (171, 172).

NK cells have also been shown to interact with neutrophils and in negatively reducing neutrophilic inflammation in the lung (191). Depletion in neutrophils influence the function and activation of NK cells in Mtb infected lungs. Increase in the NK cells following infection has been observed in Mtb infected mice, indicating a vital role of NK cells in initial innate immunity against the bacteria. NK cells have been shown to be crucial in the maturation of dendritic cells (DC), the major antigen presenting cells to T-cells either by directly interacting with the receptors on these cells or through IFNγ. NK cells enhance the proliferation of γδ T cells and Mtb specific CD8^+^ T cells, essential for anti-TB immunity (171). Additionally, NK cells cause cytotoxicity in iDCs (immature DC) and T-regs (regulatory T-cells) which suppress Mtb-specific host immunity, suggesting the role of immunoregulatory NK cells in maintaining equilibrium between the effector and regulatory branches of adaptive immunity (171).

Interestingly, NK cells express markers associated with memory upon antigen exposure, highlighting the role of NK cells in enhancing responses following vaccination. Long term NK cell protection in BCG vaccinated mice was observed by an IL-21 dependent increase in IFNγ producing memory NK cell population following Mtb challenge suggesting NK cells as an important mediator of trained immunity (167, 192, 193). Further, a recent study showed that BCG vaccinated infants had significantly more IFNg producing NK-cells (194). Additionally, most studies investigating the function of NK cells in TB have been performed in human peripheral blood or susceptible mouse models of TB that develop severe inflammatory disease. However, the role of NK cells in latent TB infections is incompletely understood. Further research is warranted to delineate the exact functions of NK cells during TB especially in the context of trained immunity to vaccines.

### 3.7 Invariant natural killer T-cells

iNKT cells are lymphocytes that exhibit characteristics of both the innate and adaptive immune systems. These cells co-express NK cell and T-cell markers. They recognize glycolipid antigens in a CD1d-restricted manner and exert immunoregulatory activities through NK cell-like cytolytic activity and rapid cytokine production (195, 196).. iNKT cells are known for their quick innate-like early response and adaptive-like memory response. The protective role of iNKT cells has been documented in various viral and bacterial infections, including influenza and *Pseudomonas aeruginosa* (197–201).During influenza, iNKT cells not only contribute to rapid early defense but also play a crucial role in modulating CD8+ T cell responses to control the infection (202). However, in airway inflammatory diseases like allergic asthma and COPD, iNKT cells can contribute to disease pathogenesis due to their ability to produce Th cytokines (195, 196, 203–206).

During TB, the frequency of iNKT cells rapidly increases in the first two weeks after TB infection but subsequently decreases as CD4 and CD8 cells take over (207). *In-vitro* studies have shown that CD1d restricted iNKT cells can effectively limit bacterial replication (208). Adoptive transfer of iNKT cells from naïve mice to Mtb-infected mice has been shown to prolong the survival of the infected mice (208). iNKT cells can be directly activated by TCR recognition of mycobacterial lipid antigens presented on the CD1d molecule of antigen-presenting cells (APCs), or indirectly activated by the release of IL-12 and IL-18 by APCs in response to antigen stimulation (208, 209). Once activated, iNKT cells promptly produce various cytokines, including IFNγ, TNFα and IL-10 which actively recruit and activate neutrophils, macrophages, NK cells, and other immune cells to the site of infection. During Mtb infection, iNKT cells also produce CD1d dependent GM-CSF, which mediates anti-mycobacterial functions (209). iNKT cells also contribute to adaptive immune functions by enhancing dendritic cell (DC) antigen presentation to T-cells and directly activating T-cell effector functions through cytokine signaling. It has been reported that during Mtb infection, iNKT cells can induce the production of IgG and IgA by B-cells through the production of IL-21 (210).

α-Galactosylceramide (αGalCer) is a well-studied CD1d ligand and is known to activate iNKT cells. Treatment of susceptible mice with αGalCer increased survival, reduced bacterial burden and immunopathology of TB disease (211, 212). Furthermore, vaccinating mice with αGalCer also protected mice from TB in an iNKT cell dependent manner (213, 214). An informative human TB patient study compared peripheral blood iNKT cells from active TB patients, latent TB patients and healthy controls and found reduced cell proliferation of iNKT cells in response to αGalCer *ex-vivo.* They further showed that this was a cell intrinsic defect owing to reduced CD1d expression, increased PD-1 expression and increased apoptosis in iNKT cells from active TB patients in comparison to latent TB or healthy control groups. This further shows the effect of Mtb on protective iNKT cells during infection (215).

In the peripheral blood of TB-infected patients, a decrease in NKT cell frequencies has been reported (215). Specifically, the double-negative (DN) iNKT subsets, which produce IFN-γ and other proinflammatory cytokines has been observed in active TB disease, while an increase in double-negative (DN) cytotoxic iNKT cell subsets, which produce suppressive, Th2 cytokines has been noted (216). These DN iNKT subsets, which are also found in patients co-infected with HIV and TB (217), associated with chronic disease and immunopathology related to infection. The frequency of iNKT cells is increased in latent TB patients in comparison to active TB patients (218). Therefore, iNKT cells exhibit dual- functions of immunoregulation and immunoactivation, and further studies are required to elucidate the precise role of these effector lymphocytes in early immunity to TB.

### 3.8 Innate lymphoid cells

ILC, or innate lymphoid cells, are a diverse group of tissue-resident effector cells that exhibit similar functions to T cells. Derived from the common lymphoid precursor like T cells, ILCs rapidly respond to infections by producing cytokines. Unlike T cells, ILCs lack antigen receptors (TCRs) and clonal selection. These cells accumulate at barrier sites, sensing stress and cytokine signals from myeloid cells at infection sites and contribute to effector cytokine production. Additionally, ILCs play a crucial role in initiating communication with myeloid cells and antigen-presenting cells like dendritic cells, thereby facilitating T cell maturation in the thymus. During lung infections, ILCs can activate both innate and adaptive immune cells.

ILCs can be broadly classified into three subsets. ILC1s produce IFN-γ and express T-bet and Eomes. They respond to IL-12, IL-15, IL-18, exhibit cytotoxic activity, and include NK cells. ILC2s are tissue-resident Th2-type cells characterized by GATA3 expression. They produce IL-5 and IL-13 and respond to epithelial cytokines such as IL-23, IL-33, and TSLP. ILC3s and lymphoid tissue-inducer cells (LTi) express RORγt and produce GM-CSF, IL-17, and IL-22. These subsets are crucial for mucosal host defense and respond to IL-1β and IL-23. Collectively, ILC subsets play roles in myeloid cell recruitment, B cell antibody production, modulation of antigen-presenting cells, and stimulation of effector T cell responses. Depending on the signals they receive, ILCs can contribute to immunopathology or tissue repair, maintaining lung homeostasis and airway barrier integrity. Despite being a minor fraction of the resident lung population, ILCs significantly impact disease outcomes (219–225).

In the context of influenza infection, a rapid increase in ILC2s has been observed. They play critical roles in combating the virus, promoting tissue repair, and facilitating airway remodeling post-infection. ILC1s and ILC3s have also shown beneficial effects during influenza infection (226–228). However, in airway allergic diseases like respiratory syncytial virus (RSV) infection, asthma, and chronic obstructive pulmonary disease (COPD), elevated ILC2s exacerbate disease pathology due to severe inflammatory reactions mediated by Th2 cytokines, leading to increased disease severity (229–231).

ILCs also possess memory-like functions, contributing to innate immunity and trained immunity (232). Studies on BCG vaccination have demonstrated vigorous recall responses in ILC1s/NK cells, akin to immune memory, protecting against Mycobacterium tuberculosis (Mtb) infection in mice. Furthermore, studies have shown an increase in ILC subsets in the lymph nodes and lungs of BCG-vaccinated mice (233, 234).Memory-like responses have also been reported in ILC2s following exposure to papain allergen, where pre-trained mice exhibited enhanced ILC2 responses upon re-sensitization to the same allergen (235). However, the implications of ILC memory in tuberculosis (TB) have not been explored extensively.

In relation to TB infection, recent studies have highlighted the protective effects of ILC subsets, although their roles in different stages of TB require further investigation. Mtb infection rapidly induces ILC accumulation in the lungs of patients (236, 237). It has been observed that circulating ILC subsets, including ILC1s and ILC3s, become depleted during active TB disease but accumulate in the lungs of both humans and mice. Specifically, ILC3 subsets are recruited to the lungs, particularly in induced bronchus-associated lymphoid tissues, in a CXCL13-CXCR5 axis-dependent manner. These subsets exert protective effects through the production of IL-17 and IL-22. Additionally, the recruitment of alveolar macrophages, neutrophils, monocytes, and other immune subsets coincides with this accumulation. Notably, ILC1s and ILC3s rebound in circulation after TB treatment. Deletion of ILC3 subsets compromises early control of Mtb, highlighting their protective role in the initial stages of infection (236). Another study reports that Mtb infection programs lung resident ILC precursors to differentiate into an ILC1 phenotype which exhibits sustained production of IFN-γ, which is crucial for anti-Mtb defense (238). Moreover, the protective nature of ILC3s and their IL-22-mediated effects in increasing survival and reducing inflammation have been observed in Mtb-infected mice with Type 2 Diabetes (239). However, further research is necessary to fully understand the implications of ILC effector functions and ILC memory in TB disease.

### 3.9 Unknown facts in innate immune cell-Mtb interactions in TB

**Figure 2:**
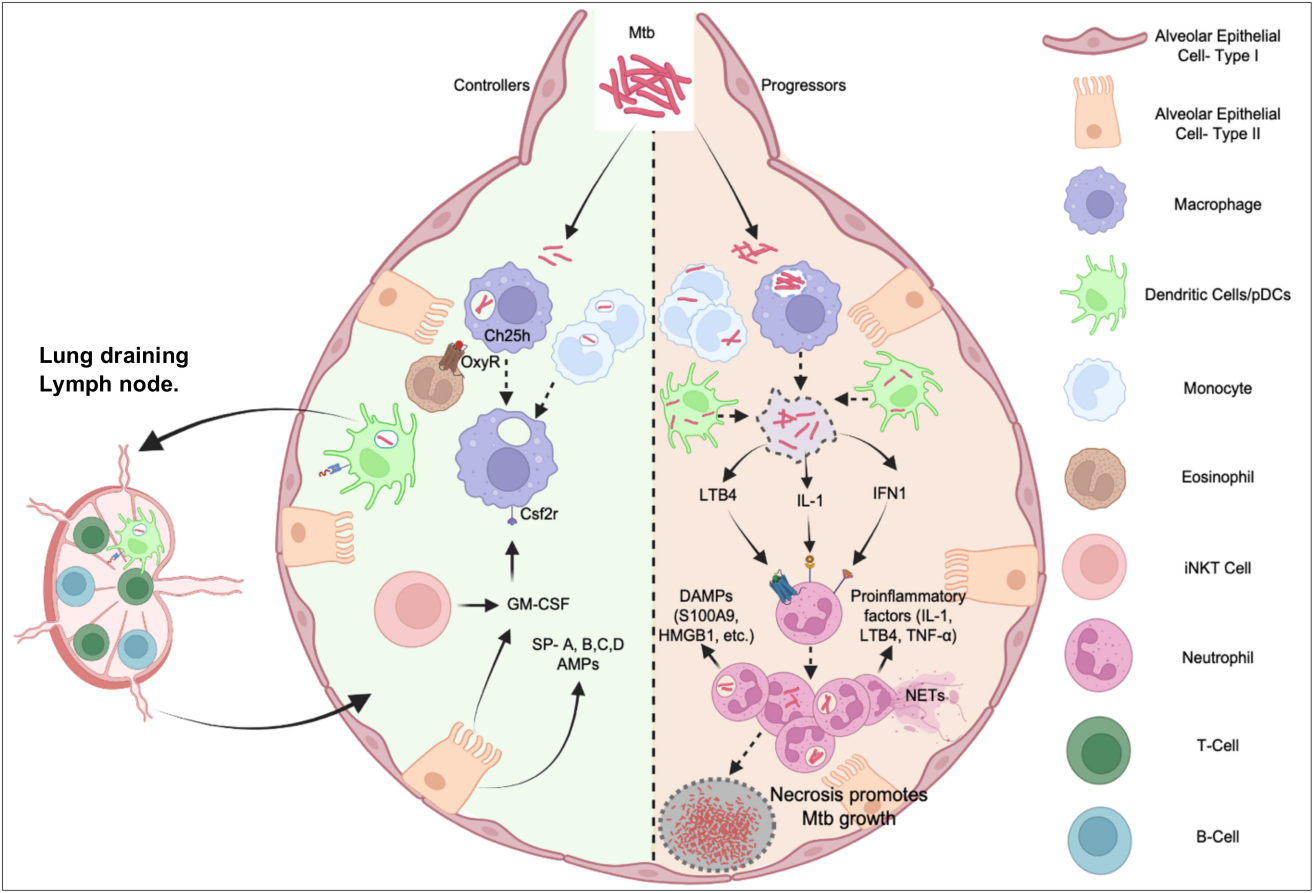
Interplay between innate immune cells in TB Disease controllers and progressors post Mtb Infection. Mtb infection of alveolar macrophages and DCs or monocytes have diverse outcome. In most individuals (Controllers) influenced by genetic and/or environmental factors, these cells kill the pathogen by poorly understood cell-autonomous mechanisms or being activated by a robust adaptive immune system to control the infection. In 5-10% cases, bacteria evade these mechanisms causing a dysregulated inflammatory response dominated by neutrophil functions, which cause immunopathology, damages the lung architecture, and generates a nutrient replete tissue microenvironment for the bacterium to multiply (Progressors). While the balance to recruit pathogen-controlling and pathogen permissive immune cells and appropriately prime adaptive immunity is critical for optimal protection, inflammatory pathways implicated in neutrophil recruitment and activation breaks the tolerance and exacerbates pathology leading to symptomatic TB disease.

While the innate immune response is crucial for tuberculosis immunity (**Figure 2**), several areas of research remain unknown and require further investigation.

#### 3.9.1 Mechanisms of bacterial recognition

The precise mechanisms by which different innate immune cells detect and recognize Mtb are not yet fully understood. Identifying the specific pattern recognition receptors involved and the signaling pathways triggered will provide valuable insights. While various pathogen recognition receptors (such as mannose, complement, Fc-receptors, Dectin-1, NOD2, NLRP3) in macrophages and DCs following Mtb exposure have been extensively studied, significant gaps exist in understanding the downstream consequences of these recognitions. It is still unclear whether different cells engage distinct recognition mechanisms for Mtb. Their ultimate impact on infection outcome, most importantly specific pathogen recognition patterns that leads to bacterial killing, as opposed to replication, and spread, remain open questions requiring investigation.

#### 3.9.2 Host-pathogen interactions

The interactions between Mtb and various innate immune cells are complex and not fully characterized. Further research is necessary to understand how the bacterium evades immune responses and manipulates innate immune signaling. Whether the initial host-pathogen crosstalk differs between hosts that control the infection and those who experience progression to active disease is still unknown.

#### 3.9.3 Immunopathology

Tuberculosis is characterized by immunopathological damage to the lungs, impairing respiratory functions. The role of different innate immune cells and their crosstalk with the lung stroma in driving or mitigating immunopathology needs to be examined. Existing evidence suggests a role of neutrophil granulocytes in addition to an early protective interaction with eosinophils. However, how this response is determined in the lung environment initially and identifying the early cellular events leading to host resistance and tolerance is key to the design of therapies and vaccines.

#### 3.9.4 Heterogeneity within innate immune cells

Innate immune cells, such as macrophages, DCs, and neutrophil granulocytes, exhibit heterogeneity, with distinct subsets possessing different functional properties. Understanding the functional diversity within these cell populations and their contributions to TB immunity is an active area of research. Eosinophils have recently emerged as granulocyte subsets to be recruited to the airways after Mtb infection, preceding neutrophils. They play a role in immune responses to infections associated with type-2 immunity. Mast cells, another type of innate cell associated with type-2 immunity, are also present in TB granulomas (240). Further research is needed to understand the roles of these granulocytes in tuberculosis pathogenesis. Additionally, distinct subsets of neutrophils with protective and pathological functions, their spatio-temporal regulation in TB granulomas may offer novel insights into TB progression and needs to be examined.

#### 3.9.5 Interaction with the adaptive immune response

With the emergence of innovative immunological tools and techniques enabling the study of immune responses at the individual cell level *in vivo*, we now recognize a vast array of functionally diverse innate and adaptive effector cells that engage in numerous cellular interactions. While the effector functions of inflammatory myeloid cells and T cells (CD4/CD8) are crucial for host protection, these cell types also possess the potential to cause tissue damage. Therefore, a key objective is to comprehend the factors that govern the delicate balance between detrimental and protective cellular immunity. Further research is necessary to elucidate the crosstalk between innate and adaptive immune cells during tuberculosis infection especially very early during infection before chronicity is established. In summary, innate immune cells play critical roles in TB immunity by initiating the response, eliminating the bacteria, and shaping the subsequent adaptive immune response. However, many aspects of the precise mechanisms and interactions involved are still unknown, emphasizing the need for ongoing research in this field.

### 3.10 Innate immune response to HIV-TB Co-infection

HIV represents one of the most significant global health challenges, with 39 million people affected by the disease in 2022 (241). TB is responsible for 32% of deaths in HIV patients, yet the immune mechanisms underlying HIV-TB comorbidity remain inadequately explored. Traditionally, it has been believed that the primary cause of increased susceptibility to TB in HIV patients is the deficiency of CD4+ T-cells, which HIV primarily targets (242–244). However, recent research sheds light on the role of innate immunity in this context and how compromised innate immune responses in HIV patients may contribute to their heightened vulnerability to Mtb infections.

As mentioned earlier, alveolar macrophages (AM) play a crucial role in interacting with Mtb and initiating innate immune responses in the lungs. In HIV patients, these AM act as reservoirs for the virus, significantly impairing their phagocytic killing capacity and cytokine production (245, 246). Consequently, these AM become more susceptible to Mtb infections and are unable to carry out their anti-mycobacterial functions effectively (247). *In vitro*studies in AMs isolated from bronchoalveolar lavage fluid (BALF) of HIV patients have demonstrated reduced transcriptional activation of macrophages compared to healthy controls (248). Conversely, Mtb infection creates a favorable microenvironment within AM for HIV survival and replication by inducing microtubule formations through a Siglec-1-IFN mediated pathway (249, 250). Furthermore, HIV-Mtb coinfection leads to an increase in the production of proinflammatory cytokines, such as IL-1β, IL-6, and GM-CSF, supporting enhanced viral replication (251, 252). Consequently, the coinfection of HIV and Mtb in AM results in a synergistic effect characterized by impaired cell phagocytosis, a shift towards necrosis over apoptosis, and increased production of proinflammatory cytokines, ultimately promoting the proliferation of both pathogens (246).

Monocytes, which are important in recruiting immune cells to the lungs are also increased in HIV/TB patients (253, 254). Increased accumulation of inflammatory CD14^+^CD16^+^ monocytes are seen in HIV/TB coinfected patients (255). HIV patients with prior exposure to TB had increased monocyte activation, contributing to TB pathogenesis (256). Increased PD-L1 expression on monocytes was seen in HIV/TB coinfected patients and this positively corelated with increased viral load, and increased plasma levels of proinflammatory cytokines (257). Research has also identified heightened diversity within monocyte subsets, which could serve as a potential indicator for individuals at risk of TB progression. This underscores the need for additional investigations into the role of monocytes as predictors of comorbidity between HIV and TB (253, 257).

The Neutrophil to Lymphocyte ratio is a frequently utilized biomarker for characterizing TB Disease. In HIV patients, where T-cell counts are significantly reduced, there is an observed increase in the neutrophil ratio (258). Multiple clinical studies have proposed employing the Neutrophil/Lymphocyte ratio as a biomarker to evaluate HIV patients who might be progressing toward TB (259, 260). Additionally, individuals with HIV infection exhibit hyperinflammatory neutrophils with compromised Mtb-killing ability, which can exacerbate immunopathology and lung damage. Interestingly, this effect is reversed with the administration of Anti-Retroviral Therapy (ART) (261). Future investigation into the role of neutrophils in HIV-TB coinfection may inform new avenues for interventions.

Studies have indicated that HIV infection disrupts the normal functioning of dendritic cells (DCs), rendering them ineffective in producing proinflammatory cytokines and impairing their ability to present antigens via MHC-II, which are both crucial for DCs’ antibacterial functions (262, 263). Recent research conducted on immature human DCs revealed that DCs coinfected with Mtb and HIV exhibited reduced expression of key costimulatory molecules, including CD40, CD80, and CD86. Consequently, HIV plays a role in hindering the presentation of Mtb antigens by DCs and displayed decreased expression of proinflammatory cytokines such as IL-6, IL-1β, and TNFα (161, 264).

Natural killer (NK) cells have well-established roles in combatting both HIV and TB infections. In cases of HIV/TB coinfection, the frequency of NK cells undergoes changes, and there is an accumulation of functionally impaired CD56-negative subsets, which can be restored through anti-TB treatment (265). Reports have also suggested alterations in the inhibitory and activation markers of NK cells, along with increased degranulation of NK cells in coinfected patients (266). Further research is needed to fully comprehend the contribution of these intermediary cells in the progression of TB in HIV-infected individuals.

Numerous reports have demonstrated the beneficial impact of ART (Antiretroviral Therapy) on innate immune cells, leading to the restoration of normal immune cell frequencies and markers after therapy (261, 267). However, a complex and paradoxical phenomenon known as Immune Reconstitution Inflammatory Syndrome (TB-IRIS) can develop in certain ART patients who are coinfected. This syndrome involves an unexpected worsening of TB symptoms and clinical manifestations following the initiation of ART. Even though the immune balance is being restored, these immune cells can become hyperactivated, leading to excessive inflammation and tissue damage (268–270). This process is primarily mediated by innate immune cells. Ideally, individuals coinfected with HIV and TB who are initially treated with anti-TB antibiotics before starting ART can potentially avoid the onset of TB-IRIS. The difficulty in modeling TB-IRIS in small animal systems contributes to the limited understanding of the pathogenesis of this inflammatory condition. Consequently, comprehending the intricate interactions among innate-immune cells and expansion of Mtb-specific pathological T-cell response is imperative for the development of therapeutic approaches aimed at mitigating TB-IRIS.

### 3.11 Innate immune response to multi drug resistant TB

Multi Drug Resistant (MDR) Tuberculosis, commonly referred to as MDR-TB, is characterized by an infection caused by a strain of Mtb that displays resistance to the primary anti-TB medications, namely, isoniazid and rifampicin. It represents a significant and formidable challenge for healthcare systems worldwide when it comes to the management and treatment of TB (271). The incidence of MDR-TB cases has been steadily increasing, with a staggering 450,000 individuals succumbing to this form of TB in 2021 alone (241). Recent research conducted in macaque monkeys demonstrated that MDR-TB infections resulted in a heightened bacterial load within the airways, in contrast to strains of Mtb that were susceptible to drug treatment. Moreover, MDR-TB strains induced an upregulation of caspase 3 expression in monocytes/macrophages and an elevation in T-cell cytokine production (272).

Macrophages infected with MDR-Mtb exhibit changes in their cellular metabolism, leading to a shift towards aerobic glycolysis (273). Research conducted on THP-1 cell lines has indicated that MDR-TB-infected macrophages demonstrate reduced TLR-2 induction and diminished production of proinflammatory cytokines (274). These infected macrophages tend to polarize towards the M2 phenotype rather than the M1 phenotype, which is observed in macrophages from MDR-TB patients (275). This is supported by studies that have found increased levels of the anti-inflammatory cytokine IL-10 in the plasma of MDR-TB patients, along with elevated levels of IFNγ and TNF-α compared to patients with drug-susceptible TB (276). MDR-TB strains display modifications in their cell wall lipid- PDIM, and overexpression of Pdim bypasses IL-1 signaling while triggering IFN-β responses. This, in turn, promotes the metabolic reprogramming of macrophages, leading to a shift towards aerobic glycolysis (277). Importantly, Mtb carrying a drug resistance-conferring single nucleotide polymorphism in *rpoB* (H445Y) can modulate host macrophage metabolic reprogramming and induce this altered cytokine response. Additionally, a study involving monocyte-derived macrophages from peripheral blood mononuclear cells of MDR-TB patients highlighted their impaired immune responses. It was demonstrated that recombinant IFN-γ could enhance the antimycobacterial function of these macrophages (278).

A recent investigation focused on MDR-Mtb strains originating from Haarlem and Latin American regions examined neutrophils isolated from healthy blood donors and revealed intriguing findings: MDR strains elicited a diminished oxidative burst in neutrophils. This reduced response was attributed to both reduced phagocytosis and diminished formation of lipid rafts, which was linked to alterations in their cell wall composition (279). Given that Mtb and MDR-Mtb have mechanisms to evade apoptotic pathways in neutrophils and macrophages, triggering apoptosis in these innate immune cells could offer valuable insights and an alternative strategy for combating MDR-TB (280). Considering the pivotal role of neutrophils in TB, future research endeavors exploring altered neutrophil functions in MDR-TB patients will be imperative for gaining a deeper understanding of disease mechanisms and developing targeted approaches against MDR-TB strains.

## 4 Conclusion

Research into the innate immunity against TB is primarily focused on disease resistance, which refers to the ability of immune cells and their effectors to eliminate the pathogen. Indeed, much of the initial TB research centered around identifying the cell autonomous mechanisms by which activated macrophages kill or halt Mtb bacteria. However, there are still significant gaps in our comprehension of resistance mechanisms when other innate cells are taken into consideration. The precise means by innate control of Mtb infection is established at the cellular level remain unclear. Moreover, the exact roles of several innate effector cells, including nonclassical T cells, NK cells, type 2 granulocytes and lung parenchymal cells that anatomically represent the very first cells in the airway to interact with the pathogen remain obscure.

Mortality due to human TB results from inflammatory destruction of the lung tissue. However, in most susceptible strains of mice that are rescued by neutrophil depletion, there is an increase in bacterial burden in the lungs, raising the question of whether a failure of resistance drives the excessive inflammation leading to death. Recently, the concept of disease tolerance, which pertains to limiting the collateral damage caused by the immune response to infection, has garnered interests of the research community. Murine studies employing streptomycin resistant Mtb strain suggests that disturbing tolerance mechanisms appears causal to host susceptibility (106, 107). Therefore, identifying early events in cellular interactions within the lung environment that leads to tolerance is key to designing intervention strategies.

Tuberculosis represents a remarkably heterogenous disease that varies within individual patient and among different patients. Developing novel therapeutics that appropriately modulate inflammation for individual patients or enhance resistance mechanisms requires a deeper understanding of the innate cells-Mtb interactions, and pathways contributing to disease progression.

## Author contributions

PS: Conceptualization, Writing – original draft, Formal Analysis. BBM: Conceptualization, Writing – review & editing, Funding acquisition.
